# Predictive Biomarkers and Patient Outcome in Platinum-Resistant (PLD-Treated) Ovarian Cancer

**DOI:** 10.3390/diagnostics10080525

**Published:** 2020-07-28

**Authors:** Isabel J. Dionísio de Sousa, Durval S. Marques, Catarina Príncipe, Raquel V. Portugal, Sule Canberk, Hugo Prazeres, José M. Lopes, Etel R. P. Gimba, Raquel T. Lima, Paula Soares

**Affiliations:** 1 Faculty of Medicine, University of Porto, 4200-319 Porto, Portugal; isajose8@gmail.com (I.J.D.d.S.); raquelvpt@hotmail.com (R.V.P.); jmlopes@ipatimup.pt (J.M.L.); psoares@ipatimup.pt (P.S.); 2 Department of Oncology, Centro Hospitalar e Universitário de João, 4200-450 Porto, Portugal; 3 Natural Science Department, Health and Humanities Institute, Fluminense Federal University, Rio das Ostras 28895-532, Brazil; dumarquesbio@hotmail.com (D.S.M.); etelgimba@id.uff.br (E.R.P.G.); 4 Cellular and Molecular Oncobiology Program, Research Coordination, National Institute of Cancer, Rio de Janeiro 20231-050, Brazil; 5 i3S—Instituto de Investigação e Inovação em Saúde, Universidade do Porto, 4200-135 Porto, Portugal; catarinas@ipatimup.pt (C.P.); scanberk@ipatimup.pt (S.C.); hprazeres@i3s.up.pt (H.P.); 6 Cancer Signalling and Metabolism Group, IPATIMUP—Institute of Molecular Pathology and Immunology, University of Porto, 4200-135 Porto, Portugal; 7 Department of Biology, Faculty of Sciences, University of Porto, 4169-007 Porto, Portugal; 8 Department of Pathology, Faculty of Medicine, University of Porto, 4200-319 Porto, Portugal

**Keywords:** ovarian cancer, tumor microenvironment, E-cadherin, vimentin, osteopontin, osteopontin-c, prognostic biomarkers, predictive biomarkers

## Abstract

Identification of predictive biomarkers for ovarian cancer (OC) treatment, particularly in the platinum-resistant/refractory setting, is highly relevant for clinical management. E-cadherin, vimentin, and osteopontin (OPN) are proteins associated with tumor microenvironment (TME) remodelling that play key roles in cancer. This study aimed to evaluate the association between the staining patterns of these proteins with survival outcomes in a series of OC patients, namely in patients with platinum-resistant/refractory disease. Low E-cadherin expression and high vimentin expression in all patient groups (as well as for E-cadherin in the platinum-resistant arm) were significantly associated with longer overall survival (OS). Low cytoplasmic OPN expression (and cytoplasmic and membrane OPN in the platinum-resistant arm) were significantly associated with longer OS. In patients that responded to treatment (pegylated liposomal doxorubicin (PLD) or other), low cytoplasmic OPN expression was also associated with longer progression-free survival (PFS). In the other hand, high nuclear OPN-c expression in patients that respond to treatment was associated with longer OS and longer PFS. Longer PFS was also associated with high expression of both nuclear and cytoplasm OPN-c, in platinum-resistant patients and in those that responded to PLD. Our study indicates that the expression of E-cadherin, vimentin, and OPN may have prognostic implications. Nuclear OPN-c and cytoplasm OPN expression are putative predictive markers in platinum-resistant (PLD treated) ovarian cancer patients.

## 1. Introduction

Ovarian cancer (OC) is the leading cause of death among all gynecological cancers in developed countries and encompasses a heterogeneous group of malignancies, with the epithelial subtype being the most frequent [1,2]. Currently, epithelial fallopian tube cancer (FTC), primary peritoneal cancer (PPC), and OC are staged and treated similarly, regardless of the site of origin [3,4]. OC high mortality/incidence ratios stem from its late diagnosis, associated with the frequent development of resistance to chemotherapy (mainly platinum compounds) [1,2]. Despite OC initial response to platinum-based chemotherapy, approximately 20% of women display disease progression within 6 months upon receiving or during a platinum-based regimen (known as platinum-resistant/refractory disease), with the majority relapsing within 12 to 18 months [2,5]. These patients present a dismal prognosis, with median survival rates ranging from three to nine months after relapse [2,5]. Monotherapy options are usually used after platinum resistance, including pegylated liposomal doxorubicin (PLD), one of the most active therapeutic options in this setting [6,7,8].

Increasing evidence indicates that several tumor microenvironment (TME) molecules correlate with disease progression and survival in OC [9]. As such, proteins associated with TME remodeling seem to be promising prognostic indicators and therapeutic targets that might be effective across a wide patient population [9]. Among TME proteins, E-cadherin, vimentin, and osteopontin (OPN) have been recently reported as associated with tumor progression and patient outcome in various cancer models.

It has been advanced that decreased E-cadherin expression plays a role in the shedding of OC cells into the abdominal cavity, which then aggregates in suspension, forming multicellular structures with differential expression levels of epithelial-mesenchymal transition (EMT) markers, namely E-cadherin and vimentin [10].

E-cadherin is a 120 kDa transmembrane protein of epithelial cells encoded by CDH1 gene [10,11]. E-cadherin presents an extracellular domain, associated with cellular adhesion, and an intracellular domain that interacts with the actin cytoskeleton strengthening cell–cell interactions, partaking signal transduction pathways [12]. E-cadherin is a reported tumor suppressor gene, and its downregulation has been identified in various cancers [13]. Several mechanisms may influence E-cadherin dysfunction, such as loss of heterozygosity at the 16q22.1 chromosome region, inactivating mutations or CDH1 gene promoter hypermethylation, overexpression of E-cadherin transcriptional repressor factors, and post-translational modifications (i.e., phosphorylation and glycosylation) [14]. Previous studies reported associations between low E-cadherin (total and membrane) levels and OC poor prognosis [10,15].

Vimentin is a member of the intermediate filament family and a cytoskeleton protein ubiquitously expressed in mesenchymal cells [16]. It has been considered a canonical marker of epithelial–mesenchymal transition (EMT) [17], and its increased expression was reported in a wide range of cancers, including gastrointestinal, prostate, breast, central nervous system (CNS), lung, and malignant melanoma [18,19,20,21]. Concerning vimentin expression and its relation to drug resistance in OC cells, a study by Kanakkanthara et al. [22] reported that it was downregulated in cisplatin-resistant cells and OC cells with acquired resistance to tubulin-targeting drugs. These data suggested that vimentin silencing has an important role in drug resistance in OC.

Osteopontin (OPN) is an extracellular matrix glycoprotein, involved in several physio-pathological processes, including tumorigenesis [23]. OPN is usually overexpressed in OC and other types of cancers [24,25,26]. OPN primary transcript is subject to alternative splicing and, in addition to other post-transcriptional and post-translational OPN variants, total OPN (the sum of all OPN variants, which we named herein as OPN) has been widely studied in cancer cells [27]. Among OPN splice variants, Osteopontin-c (OPN-c) splicing isoform seems to activate OC progression features [28]. The role of OPN and OPN-c in OC has been intensively studied in two recent published meta-analyses [29,30], indicating that it may be a potential diagnostic and prognostic biomarker. Another study reported that OPN levels were increased in advanced FIGO stages of OC [31,32], suggesting that the diagnostic sensitivity of OPN might be higher in advanced stages [33]. Although there is mounting evidence suggesting that OPN and OPN-c might be potential OC diagnostic and prognostic biomarkers as in other tumor types, prospective studies are needed to clarify the role of OPN in OC, specifically as a predictive or prognostic biomarker [27,32]. Moreover, some reports suggest a role for OPN in regulating the expression of mesenchymal-type and epithelial-type markers, such as vimentin and E-cadherin [34,35].

This study aimed to evaluate the association between the immunohistochemical staining of E-cadherin, vimentin, OPN, and OPN-c splice variant with survival outcomes in a series of patients with OC, with a specific focus on platinum-resistant/refractory disease.

## 2. Materials and Methods

### 2.1. Patients

This retrospective study used samples from patients from the Portuguese Oncology Institute of Coimbra (IPO-C). All patient clinical records and sample were obtained under approval by the institutional Ethical Committee (nr 12/TI/14, 24 September 2014), with no patient consent being required given the retrospective design of the study. Eligible patients were older than 18 years with histologically diagnosed sporadic OC, FTC, or PPC between January 2009 and December 2015, and designated as OC from now on given the similar staging and treatment, regardless of the site of origin. Sixty-eight patients were selected, with a median follow-up of 38 months (5–91). From these, 37 patients had received treatment with PLD in the context of platinum-resistant disease.

### 2.2. Tissue Samples

Formalin-fixed, paraffin-embedded tissues, and clinical data were retrieved from patients with OC at the Portuguese Institute of Oncology, Coimbra (IPO-C). The diagnosis of OC was revised by two pathologists without knowledge of clinical and follow-up features. The histologic subtypes, after histological revision, are presented in Section 3.1. Clinicopathological and molecular variables, as well as follow up data, were obtained from the surgical pathology reports and patients’ records from the Department of Pathology and Oncology of IPO-C databases. Immunohistochemical expression analysis was performed on consecutive OC tissue sections from 49 cases.

### 2.3. Evaluation of the Immunohistochemical Expression of E-cadherin, Vimentin, and Osteopontin

Immunohistochemistry (IHC) staining procedures were carried out on representative tumor tissue sections from 98 formalin-fixed, paraffin-embedded blocks. Slides were deparaffinized twice in xylene for 10 min and rehydrated in decreasing ethanol steps, followed by a single wash for 5 min in phosphate buffered saline (PBS) containing Tween 0.01% (PBS/Tween20) for 10 min at room temperature. Antigen retrieval conditions varied according to each antigen tested, which are shown in Table 1. Then, endogenous peroxidase was blocked for 10 min in 3% hydrogen peroxide dissolved in methanol, followed by two consecutive washes in PBS/T20, 5 min each. Avidin block was subsequently performed for 10 min using 100 μL of Avidin Block (REF. TA-015-BBA, Thermo scientific, Freemont, CA, USA), followed by two 5 min washes in PBS/T20. Next, endogenous biotin was blocked for 10 min at room temperature with 100 μL of Biotin Block Solution (REF. TA-015-BBB, Thermo scientific), for two further 5 min washes in PBS/T20. Tissues samples were afterwards blocked for 10 min at room temperature with 100 μL of UltraVision Protein Block (REF. TA-125-PB, Thermo Scientific). Then, slides were incubated with each corresponding primary and secondary antibodies and the conditions are shown in Table 1.

After primary and secondary antibody incubations, slides were washed for 5 min, three times in PBS/T20. Chromogenic visualization was performed incubating the slides for 10 min at room temperature with streptavidin peroxidase (REF. TS-125-HR, Thermo Scientific), followed by two washes in PBS/T20. Then, 3,3′-Diaminobenzidine (DAB) (REF. K3468, Dako, Carpinteria, CA, USA) was added and incubated for 3 min and slides were washed in water for 5 min. Slides were counterstained with Mayer’s Haematoxylin (Thermo Scientific), washed, dehydrated, cleared in xylene, and mounted in Richard–Allan Scientific^TM^ Mounting Medium. All immunohistochemical staining included gallbladder tissue sections used as positive controls, as well as negative controls by omitting the primary antibody. Immunohistochemical expression analysis was performed on consecutive OC tissue sections of a set of 49 specimens. Slides were blindly evaluated (S.C. and R.P.) according to the percentage of tumor-stained cells, intensity, and cellular localisation of the staining. Photographs were acquired using Nikon DS-L1 camera in 200× magnifications. The same tissue set was used to evaluate the expression of E-cadherin, vimentin, OPN, and OPN-c.

Semiquantitative expression analysis was evaluated using a staining score previously established [36]. Proportion of positive-stained tumor cells was scored as < 5% = 0, 5–25% = 1, 25–50% = 2, 50–75% = 3, and >75% = 4, while staining intensity values were classified as absent = 0, faint = 1, moderate = 2, or strong = 3. The established staining score (ranging from 0 to 7) corresponds to the sum of staining intensity and proportion of positive-stained tumor cells (Tables 2 and 3). For OPN and OPN-c evaluation, additional scores were used in some analysis, consisting of the sum of the score obtained for the cytoplasm and membrane staining for OPN and in the sum of cytoplasm and nuclear staining for OPN-c.

### 2.4. Survival Endpoints

Progression-free survival (PFS) was considered as the time from treatment initiation to the date of disease progression, death from any cause, or date of last follow-up. Overall survival (OS) was considered as the interval between treatment initiation and death from any cause or date of last follow-up.

### 2.5. Clinical and Radiological Assessments

Disease assessment was performed according to clinical practice and staging determined by the International Federation of Gynaecology and Obstetrics (FIGO) system [2]. Performance status (PS) was evaluated following the World Health Organization (WHO) criteria [37]. The response rate was evaluated using Response Evaluation Criteria in Solid Tumours (RECIST) 1.1 [38].

### 2.6. Statistical Analysis

Statistical analysis was performed using 26.0 SPSS statistical package. χ^2^ and independent samples *t*-test were performed to verify association(s) between E-cadherin, vimentin, OPN, and OPN-c expressions and development of platinum-resistant/refractory disease. Linear regression was used to study the relationship between OPN and OPN-c levels of expression with those of E-cadherin and vimentin. Kaplan–Meier method was used to estimate PFS and OS. The stratified log-rank test was used to compare curves between the groups (E-cadherin, vimentin, OPN, OPN-c). The significance threshold was a two-sided *p*-value of 0.05.

## 3. Results

### 3.1. Patient Sample Features

A retrospective analysis of patients’ clinical records from a single institution with histologically diagnosed OC was performed between January 2009 and December 2015.

With a median follow-up of 38 months (5–91 months), a total of 68 patients were selected, among which 37 patients had received PLD treatment (PLD arm), in the context of platinum-resistant disease. The median age was 57.0 years (39–79) in all the series and 56.5 years (39–73) in the PLD arm. From the 37 PLD-treated patients, 21 of them responded and 16 progressed. The baseline clinic characteristics of the patients were similar in the two groups (Table 2) Additional data regarding tumor samples analyzed are included in Supplementary Materials.

### 3.2. Immunohistochemistry Analysis

**Figure 1:**
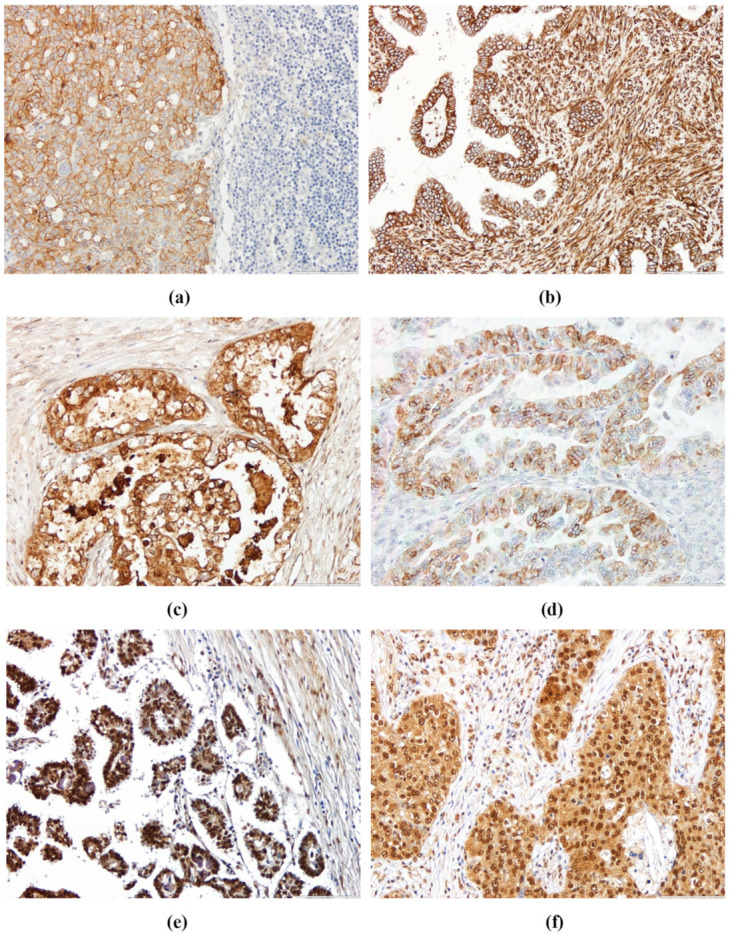
Representative sections of ovarian cancer (OC) samples showing different staining patterns for (**a**) E-cadherin, (**b**) vimentin, (**c**) osteopontin (OPN) cytoplasm, (**d**) OPN membrane, (**e**) nuclear OPN-c, and (**f**) cytoplasm OPN-c expression (200× magnification).

E-cadherin, vimentin, OPN, and OPN-c immunohistochemical expression was analyzed in the series (in 49/68 samples, as no additional material was available for some cases). Representative images of IHC staining for E-cadherin, vimentin, OPN, and OPN-c are shown in Figure 1.

E-cadherin was mainly identified at the cell membrane (Figure 1a). Vimentin presented stain at the membrane and at the cytoplasm (Figure 1b). OPN staining was located in the cytoplasm, cell membrane, and in dispersed nuclei, but only cytoplasm and membrane staining were evaluated (Figure 1c,d). OPN-c staining was observed at the cell cytoplasm and in the nucleus, both of which were considered for staining score analysis (Figure 1e,f).

The detailed results of IHC are presented in Table 3 and Table 4.

In most of the analyzed cases, a high proportion presented E-cadherin stained tumor cells (>75%) and moderate/strong staining intensity. For vimentin, a low proportion of positively stained tumor cells (<5%), and a negative or low staining score was observed in the majority of the cases. In the positively stained samples, the staining intensity was predominantly strong. Concerning cytoplasm and membrane OPN staining, most of the cases presented a low proportion of positively stained tumor cells (<5%) and a negative or low staining score. Nuclear OPN-c was positively stained in >75% of tumor cells in a high proportion of cases, with a staining score predominantly ≥4. For cytoplasm OPN-c, a high proportion of cases presented a low proportion of positively stained tumor cells (<5%), most of which had a low or negative staining score.

The results obtained in the non-PLD arm in the proportion of positively stained cells, staining intensity, and score for all proteins are described in Supplementary Table S1.

### 3.3. E-cadherin, Vimentin, OPN, and OPN-c Staining Patterns in OC Cases and Survival Outcomes

In all the series of OC analyzed, the median PFS was 13 months (3–86 months) with a median OS of 21 months (1–91 months).

**Figure 2:**
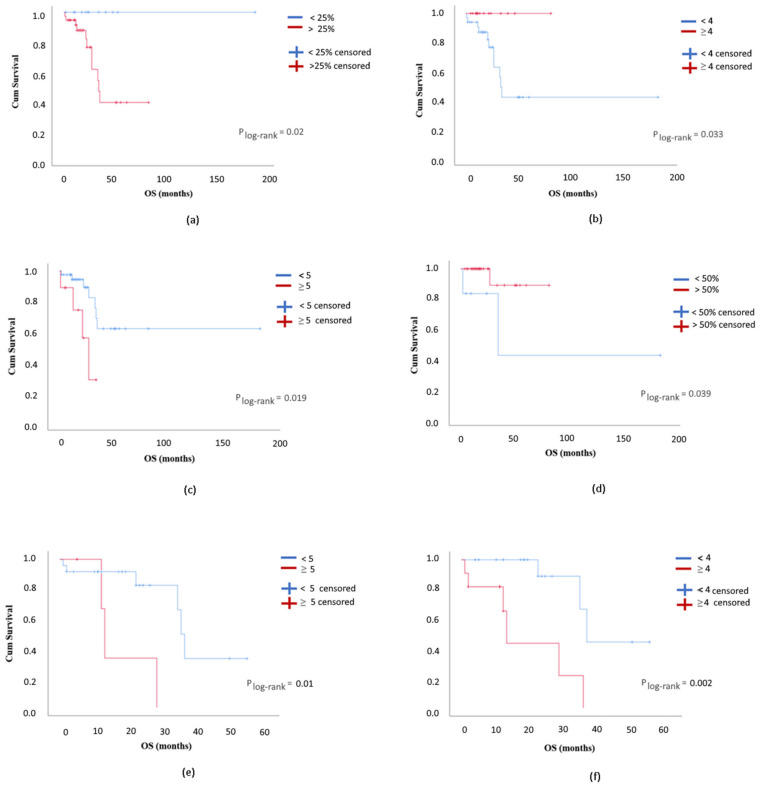
Overall survival (OS) of ovarian cancer patients according to (**a**) the proportion of positive stained tumor cells for E-cadherin (<25% vs. >25%; *n* = 49); (**b**) vimentin staining score (<4 vs. ≥4; *n* = 49); (**c**) cytoplasm OPN staining score (<5 vs. ≥5; *n* = 49); (**d**) the proportion of positively stained tumor cells for nuclear OPN-c in responders (<50% vs. >50%; *n* = 33); (**e**) membrane OPN staining score in the pegylated liposomal doxorubicin (PLD) arm (<5 vs. ≥5; *n* = 28); and (**f**) cytoplasm OPN-c staining score in the PLD arm (<4 vs. ≥4; *n* = 28).

In all the OC series, a proportion of E-cadherin positive stained tumor cells <25% significantly associated with longer OS (*p* = 0.02; Figure 2a). Concerning vimentin, the proportion of positively stained tumor cells > 5% and staining score ≥4 significantly associated with longer OS (*p* = 0.024 and 0.033, respectively; Figure 2b). Considering cytoplasm OPN, a proportion of positively stained tumor cells <25% and a staining score <5 were associated with a significant longer OS (*p* = 0.037 and 0.019, respectively; Figure 2c), whereas membrane OPN staining pattern was not significantly associated with either PFS or OS. A combined staining score for cytoplasm and membrane OPN revealed a significant association between low staining score (<8) and longer PFS (*p* = 0.037).

For responders to treatment in all the series, no significant associations were observed between PFS or OS and the proportion of positively stained tumor cells, as well as the intensity of staining (or staining score) for E-cadherin, vimentin, membrane OPN, or cytoplasm OPN-c. Only for cytoplasmic OPN, a proportion of positively stained tumor cells <25% and a staining score <4 associated with a significant longer PFS (*p* = 0.047 for both criteria). A low combined staining score for cytoplasm and membrane OPN (<8) was associated with a significant longer PFS (*p* = 0.029) in patients that responded to therapy. For nuclear OPN-c, a staining score ≥1 was associated with a significant longer PFS (<0.001), while a proportion of positive stained tumor cells >50% was associated with a significantly longer OS (*p* = 0.039; Figure 2d).

Considering the platinum-resistant patients treated with PLD (PLD arm), a median PFS of 10 months (3–26 months) with a median OS of 35 months (3–73 months) was observed.

In the PLD-treated arm, a proportion of positive tumor cells <25% and a staining score <5 for E-cadherin were associated with a longer OS (*p* = 0.015 and 0.008, respectively). Concerning cytoplasm and membrane OPN, a proportion of positive tumor cells <25% and a staining score <5 significantly associated with longer OS (cytoplasm OPN *p* = 0.003 and 0.017, respectively; membrane OPN *p* = 0.01 for both; Figure 2e). A combined staining score for cytoplasm and membrane OPN revealed a significant association between low staining score (<5) and longer OS (*p* = 0.005). For nuclear OPN-c, a staining score ≥5 was associated with a significant longer PFS (*p* < 0.001). A proportion of positive tumor cells >5% and a staining score ≥ 1 for cytoplasmic OPN-c were associated with a significantly longer PFS (*p* = 0.002 and *p* = 0.001, respectively). A cytoplasm OPN-c proportion of positive tumor cells < 25% and a staining score <4 were associated with a longer OS (*p* = 0.01 and 0.002, respectively; Figure 2f). A combined staining score for nuclear and cytoplasm OPN-c revealed a significant association between a high staining score (≥5) and longer PFS (*p* < 0.001), while a staining score <8 was significantly associated with an improvement in OS (*p* = 0.01).

Regarding the responders’ group in the PLD arm, no statistically significant associations were observed between PFS or OS and the proportion of positively stained tumor cells, the intensity of staining, or staining score for E-cadherin, vimentin, or membrane OPN. For cytoplasm OPN, a proportion of positively stained tumor cells <50% was associated with a significantly longer PFS (*p* = 0.037). Concerning nuclear OPN-c, a staining score ≥5 was associated with a significantly longer PFS (*p* < 0.001). Cases with a cytoplasm OPN-c with a proportion of positively stained tumor cells >5% and a staining score ≥1 disclosed a significantly longer PFS (*p* = 0.005 for all criteria). Cases with a cytoplasm OPN-c staining score <4 presented a significantly longer OS (*p* = 0.02). A combined staining score for nuclear and cytoplasmic OPN-c ≥ 5 was significantly associated with a longer PFS (*p* < 0.001).

Additionally, we found that, in all the series, cytoplasm OPN-c was associated with an increase in E-cadherin staining score (*p* = 0.006) and cytoplasmic OPN with an increase in vimentin staining score (*p* = 0.01). In the responder’s group, nuclear and cytoplasm OPN-c was associated with an increase in E-cadherin staining score (*p* = 0.05 and 0.027, respectively) and cytoplasm OPN with an increase in vimentin staining score (*p* = 0.01).

## 4. Discussion

Platinum-resistant/refractory disease occurs in approximately 20% of women and is an important setting in OC patients’ management. In this situation, PLD has an important role in OC therapy [39,40]. Furthermore, there is a need for better predictive biomarkers in OC, especially in the platinum-resistant/refractory disease. This work addresses this need by evaluating the expression of the TME-associated proteins, E-cadherin, vimentin, and OPN, and its possible association with OC patient’s survival outcomes.

In this study, low immunohistochemical staining for E-cadherin in all the series and in the PDL arm was associated with longer OS. These results seem to be in agreement with others in which higher E-cadherin (although analyzed at the mRNA level) was associated with shorter platinum-free progression intervals (less than 6 months) and higher levels of cancer antigen 125 [10]. The relationship between E-cadherin levels and OC progression, dissemination, and aggressiveness is still controversial [10], with both high and low E-cadherin expression levels being reported [10,41]. Yet, low E-cadherin expression observed in advanced-stage tumors might favour OC dissemination by direct extension of tumor cells into the peritoneal cavity. Further research is needed to clarify the role of E-cadherin in the different OC settings.

Concerning vimentin, we observed that a higher immunohistochemical expression was associated with a significantly longer OS. A study by Szubert et al. [42] also reported high vimentin expression associated with an improved OS in patients with OC, which is in line with our findings. Preclinical studies have postulated evidence indicating that increased vimentin expression is associated with platinum sensitivity, which suggests a favourable prognosis [16]. However, other studies indicate that a high expression of vimentin is associated with a poor prognosis in other cancer types, with the information on the expression of vimentin by tumor cells and prognosis in OC being scarce.

Concerning cytoplasmic and membrane OPN, in PLD arm and in the OC series (here only for cytoplasm OPN), low immunohistochemical expression levels are associated with a significantly longer OS in our study. Moreover, low immunohistochemical expression levels of cytoplasmic OPN in responders in the OC series and responders to PLD were associated with a significantly longer PFS. A low combined staining score for cytoplasm and membrane OPN was significantly associated with an improvement in PFS in the whole series and in the PLD-responders in all series and with a significant longer OS in the PLD arm. These findings seem to be in agreement with the published studies concerning the role of OPN in other tumors models, such as thyroid and breast cancer, where high expression of OPN is associated with poor prognosis [43,44].

Considering nuclear OPN-c, a higher staining score was associated with a significantly longer PFS in the PLD arm. Notably, a high immunohistochemical nuclear expression of OPN-c in the responders’ group in all the series and in the PLD arm also associated with a significant longer PFS. Accordingly, a high proportion of positively stained tumor cells was significantly associated with longer OS in patients that responded to PLD therapy in the OC series. These results suggest that OPN-c high nuclear staining might be a possible indicator of response to therapy, in both the setting of PLD therapy and other chemotherapies.

Concerning cytoplasmic OPN-c, we observed variable results. Higher immunohistochemical expression was associated with a significantly longer PFS in PLD and in responders from the PLD arms. Conversely, a low immunohistochemical expression was associated with a significantly longer OS in the PLD and responders to PLD arms. Similar associations were found when we used a combined staining score for nuclear and cytoplasmic OPN-c. As such, high immunohistochemical expression was associated with a significantly longer PFS in the PLD and responders in the PLD arms. Conversely, low immunohistochemical expression was associated with a significantly longer OS in the PLD and responders in the PLD arms.

The biological/clinical function of OPN-c in cancer, specifically in OC, has been under investigation [45]. A study by Tilli et al. [46] evaluated the expression profile of OPN isoforms in ovarian tumor and non-tumor samples in which OPN-c seems to influence the physiopathology of OC progression and tumorigenesis. Another study in breast cancer reported that high levels of OPN-c, without stating its subcellular location, appeared to be significantly associated with poor survival [47]. In our study, we found an association between nuclear OPN-c and better patient outcome, while the cytoplasmic localisation of the protein seems to have the reverse effect in some patients. We noted a tendency to an exclusive localisation of the protein either in the nucleus or in the cytoplasm. Thus, we speculate that different functions might be associated with nuclear and cytoplasmic OPN-c. Additional studies are needed to clarify the underneath mechanism by which OPN and its isoforms exert their effect and the different cellular compartments involved.

Although being the leading cause of death for gynaecological cancers in developed countries, OC is a relatively rare type of cancer and, consequently, the recruitment of large series to have robust data in treatment modalities is warranted. The number of patients, as well as its retrospective design, is an obvious limitation of our study. Nevertheless, information on the immunohistochemical expression (and subcellular localization) of the studied proteins in OC, particularly in platinum-resistant/refractory ovarian cancer patients, is still scarce and our study can contribute to further expanding this knowledge regarding the potential application of these biomarkers in the clinical setting.

## 5. Conclusions

In conclusion, the present study evaluates the expression of the TME-associated proteins, E-cadherin, vimentin, and osteopontin, in OC patients subjected to chemotherapy (including PLD). Our results point towards a possible role of E-cadherin, vimentin, and OPN as prognostic indicators in patients with OC, as evaluated by PFS and OS outcomes. Moreover, the OPN-c nuclear expression seems to be a promising marker of response to therapy (PLD or other chemotherapy) in OC patients.

## Figures and Tables

**Table 1:** Antigen retrieval and antibody experimental used conditions at immunohistochemistry.

Antigen Retrieval	Primary Antibody	Secondary Antibody
Not performed	Chicken Anti-OPNc IgY;	Goat Anti-Chicken IgY (H + L) Biotin Conjugated
	Ref # AhOPN-c NA, Gallus Immunoyech INc;	REF #A16058, NOVEX–LIFE TECHNOLOGIES^®^,
	Diluted 1:900 in antibody diluent Solution (Thermo Scientific Quanto,	Diluted 1:3000 in antibody diluent Solution (Thermo Scientific Quanto,
	Ref #TA-125-ADQ) for 30 min at RT	REF.# TA-125-ADQ) for 16 min
Antibody retrieval solution (Novocastra™-Leica Biosystems, Ref # RE 7119) 40 min at the steamer cooker, followed by 30 min at RT	Goat anti- E-cadherin	Biotinylated Goat Polyvalent
	Ref#3195 Cell Signaling)	(Ref # TP 125-BNE, Thermo Scientific,
	Diluted 1:100 Dilution1:900 in antibody diluent Solution (Thermo Scientific Quanto, Ref # TA-125-ADQ)	Solution ready to use, 10 min, RT
	for 1 h at RT	
Antibody retrieval solution (Novocastra™-Leica Biosystems, Ref # RE 7119) 40 min at the steamer cooker, followed by 30 min at RT	Monoclonal mouse anti-Vimentin	Biotinylated Goat Polyvalent
	Ref # V6389, SIGMA-ALDRICH^®^	Ref # TP 125-BNE, Thermo Scientific
	Diluted 1:400 in antibody diluent Solution (Thermo Scientific Quanto,	
	REF.# TA-125-ADQ) for 1h at RT	Solution ready to use, 10 min at RT
Citrate Buffer pH = 6 at microwave oven for 10 min, then 30 min at RT	Goat anti-total OPN;	Rabbit anti-goat
	Ref # AF1433, R&D Systems;	Ref # E0466, Dako
	Diluted 1:450 in antibody diluent Solution Thermo Scientific Quanto, REF. TA-125-ADQ) for 16–18 h at 4 °C	Diluted 1:200 in antibody diluent Solution (Thermo Scientific Quanto, REF. TA-125-ADQ) for 16 min

OPN-c: osteopontin-c; Ig: immunoglobulin; ADQ: antibody diluent OP Quanto; RT: room temperature.

**Table 2:** Summary of ovarian cancer (OC) patients’ clinical data.

	OC Series (*n* = 68)	PLD Arm (*n* = 37)
**Median age at diagnosis (years)**	57.0 (39–79)	56.5 (39–73)
ECOG PS * at diagnosis		
**0**	29 (42.6%)	13 (35.1%)
**1**	39 (57.4%)	24 (64.9%)
**Stage at diagnosis**		
**II**	14 (20.6%)	5 (13.5%)
**III**	48 (70.6%)	28 (75.7%)
**IV**	6 (8.8%)	4 (10.8%)
**Lymph node positive**	13 (19.1%)	10 (27.0%)
**Laterality**		
**Unilateral**	41 (60.3%)	20 (54.1%)
**Bilateral**	27 (39.7%)	17 (45.9%)
**Histologic subtype**		
**High-grade serous carcinoma**	37 (54.4%)	22 (59.5%)
**Low-grade serous carcinoma**	18 (26.5%)	8 (21.6%)
**Mucinous**	5 (7.4%)	3 (8.1%)
**Clear cells**	4 (5.9%)	2 (5.4%)
**Endometrioid**	4 (5.9%)	2 (5.4%)
**Initial surgery**	62 (91.2%)	33 (89.2%)
**Adjuvant chemotherapy**	62 (91.2%)	33 (89.2%)
**Therapy response**	48 (70.6%)	21 (56.8%)

* ECOG PS–Eastern Cooperative Oncology Group Performance Status; PLD, pegylated liposomal doxorubicin; OPN: osteopontin; OPN-c: osteopontin-c.

**Table 3:** IHC staining intensity and proportion of positive stained tumor cells for E-cadherin, vimentin, OPN and OPN-c in OC.

Cases Analyzed (*n* = 49)	E-cadherin	Vimentin	Cytoplasm OPN	Membrane OPN	Nuclear OPN-c	Cytoplasm OPN-c
**Proportion of positive stained cells**						
<5%	7 (14.3%)	30 (61.2%)	28 (57.1%)	37 (75.5%)	2 (4.1%)	23 (47.0%)
5–25%	5 (10.2%)	11 (22.4%)	6 (12.2%)	3 (6.1%)	5 (10.2%)	4 (8.2%)
25–50%	5 (10.2%)	2 (4.1%)	6 (12.2%)	4 (8.2%)	3 (6.1%)	5 (10.2%)
50–75%	7 (14.3%)	1 (2.0%)	5 (10.2%)	4 (8.2%)	7 (14.3%)	8 (16.3%)
>75%	25 (51.0%)	5 (10.2%)	4 (8.2%)	1 (2.0%)	32 (65.3%)	9 (18.4%)
**Staining intensity**						
Absent	5 (10.2%)	21 (42.9%)	17 (34.7%)	29 (59.2%)	2 (4.1%)	21 (42.9%)
Faint	8 (16.3%)	1 (2.0%)	13 (26.5%)	2 (4.1%)	17 (34.7%)	16 (32.7%)
Moderate	16 (32.7%)	8 (16.3%)	14 (28.6%)	5 (10.2%)	15 (30.6%)	9 (18.4%)
Strong	20 (40.9%)	19 (38.8%)	5 (10.2%)	13 (26.4%)	15 (30.6%)	3 (6.1%)
**Staining Score**						
0	5 (10.2%)	21 (42.9%)	17 (34.7%)	29 (59.2%)	2 (4.1%)	21 (42.9%)
1	1 (2.0%)	0 (0.0%)	8 (16.3%)	2 (4.1%)	0 (0.0%)	2 (4.1%)
2	3 (6.1%)	5 (10.2%)	3 (6.1%)	2 (4.1%)	5 (10.2%)	3 (6.1%)
3	3 (6.1%)	7 (14.3%)	8 (16.3%)	5 (10.2%)	0 (0.0%)	4 (8.2%)
4	7 (14.3%)	9 (18.4%)	4 (8.2%)	3 (6.1%)	6 (12.2%)	6 (12.2%)
5	5 (10.2%)	1 (2.0%)	4 (8.2%)	4 (8.2%)	9 (18.4%)	8 (16.3%)
6	8 (16.3%)	2 (4.1%)	4 (8.2%)	3 (6.1%)	12 (24.5%)	2 (4.1%)
7	17 (34.7%)	4 (8.2%)	1 (2.0%)	1 (2.0%)	15 (30.6%)	3 (6.1%)

**Table 4:** IHC staining intensity and proportion of positive stained tumor cells for E-cadherin, vimentin, OPN, and OPN-c in the PLD-treated OC.

Cases of the PLD Arm Analyzed (*n* = 28)	E-cadherin	Vimentin	Cytoplasm OPN	Membrane OPN	Nuclear OPN-c	Cytoplasm OPN-c
**Proportion of positive stained cells**						
**<5%**	4 (14.3%)	17 (60.7%)	15 (53.6%)	22 (78.6%)	1 (3.6%)	14 (50.0%)
**5–25%**	4 (14.3%)	6 (21.4%)	3 (10.7%)	2 (7.1%)	3 (10.7%)	1 (3.6%)
**25–50%**	0 (0.0%)	1 (3.6%)	4 (14.3%)	2 (7.1%)	3 (10.7%)	4 (14.3%)
**50–75%**	5 (17.9%)	0 (0.0%)	3 (10.7%)	2 (7.1%)	2 (7.1%)	6 (21.4%)
**>75%**	15 (53.6%)	4 (14.3%)	3 (10.7%)	0 (0.0%)	19 (67.9%	3 (10.7%)
**Staining intensity**						
**Absent**	2 (7.1%)	13 (46.4%)	10 (35.7%)	15 (53.6%)	1 (3.6%)	12 (42.9%)
**Faint**	6 (21.4%)	0 (0.0%)	8 (28.6%)	2 (7.1%)	10 (35.7%)	8 (28.6%)
**Moderate**	6 (21.4%)	4 (14.3%)	7 (25.0%)	2 (7.1%)	9 (32.1%)	7 (25.0%)
**Strong**	14 (50.0%)	11 (39.3%)	3 (10.7%)	9 (32.1%)	8 (28.6%)	1 (3.6%)
**Staining Score**						
**0**	2 (7.1%)	13 (46.4%)	10 (35.7%)	15 (53.6%)	1 (3.6%)	12 (42.9%)
**1**	1 (3.6%)	0 (0.0%)	5 (17.9%)	2 (7.1%)	0 (0.0%)	2 (7.1%)
**2**	2 (7.1%)	1 (3.6%)	0 (0.0%)	1 (3.6%)	3 (10.7%)	1 (3.6%)
**3**	2 (7.1%)	5 (17.9%)	5 (17.9%)	5 (17.9%)	0 (0.0%)	2 (7.1%)
**4**	3 (10.7%)	4 (14.3%)	1 (3.6%)	1 (3.6%)	3 (10.7%)	4 (14.3%)
**5**	3 (10.7%)	1 (3.6%)	3 (10.7%)	2 (7.1%)	7 (25.0%)	5 (17.9%)
**6**	4 (14.3%)	1 (3.6%)	4 (14.3%)	2 (7.1%)	6 (21.4%)	1 (3.6%)
**7**	11 (39.3%)	3 (10.7%)	0 (0.0%)	0 (0.0%)	8 (28.6%)	1 (3.6%)

OPN: osteopontin; OPN-c: osteopontin-c.

## Supplementary Materials

The following are available online at https://www.mdpi.com/2075-4418/10/8/525/s1, Supplementary Data: Additional data regarding tumor samples analyzed; Table S1: Staining intensity and proportion of positive stained cells of E-cadherin, vimentin, OPN, and OPN-c IHC in the non-PLD arm.
