# Supplementary material for: Predictive Biomarkers and Patient Outcome in Platinum-Resistant (PLD-Treated) Ovarian Cancer
Source: Diagnostics (Basel). 2020 Jul 28;10(8):525. doi: 10.3390/diagnostics10080525 (PMC7459532; doi:10.3390/diagnostics10080525)
Supplement: Supplementary file 1 [file diagnostics-10-00525-s001.pdf]

diagnostics

MDPI

Article

Predictive Biomarkers and Patient Outcome in Platinum-Resistant (PLD-Treated) Ovarian Cancer

Isabel J. Dionísio de Sousa [1,2], Durval S. Marques [3,4], Catarina Príncipe [5,6,7], Raquel V. Portugal [1,8], Sule Canberk [5,6], Hugo Prazeres [5,6], José M. Lopes [1,5,6,8], Etel R.P. Gimba [3,4], Raquel T. Lima[1,5,6,8,*], and Paula Soares [1,5,6,8]

Supplementary Data Additional data regarding tumor samples analyzed.

"Non-high grade serous carcinoma"—32% (9/28) and 48% (10/21) in PLD and non-PLD arms, respectively.

"Low-grade serous carcinoma"—18% (5/28) and 33% (7/21) of the cases in the PLD and non-PLD groups, respectively.

Among responders, in the PLD arm, 1 case was non-high grade serous carcinoma (endometrioid carcinoma) and, in the non-PLD group, 8 were non-high grade serous carcinoma (5 low- grade serous, 2 clear cell and 1 mucinous carcinoma).

Table S1. Staining intensity and proportion of positive stained cells of E-cadherin, vimentin, OPN, and OPN-c IHC in the non-PLD arm.

Non-PLD Arm. Analysed (*n* = 21)	E-cadherin	Vimentin	Cytoplasmic OPN	Membrane OPN	Nuclear OPN-c	Cytoplasmic OPN-c
**Proportion of positive stained cells**						
< 5%	3 (14.3%)	13 (61.9%)	13 (61.9%)	15 (71.4%)	1 (4.8%)	9 (42.9%)
5–25%	1 (4.8%)	5 (23.8%)	3 (14.3%)	1 (4.8%)	2 (9.5%)	3 (14.3%)
25–50%	5 (23.8%)	1 (4.8%)	2 (9.5%)	2 (9.5%)	0 (0.0%)	1 (4.8%)
50–75%	2 (9.5%)	1 (4.8%)	2 (9.5%)	2 (9.5%)	5 (23.8%)	2 (9.5%)
> 75%	10 (47.6%)	1 (4.8%)	1 (4.8%)	1 (4.8%)	13 (61.9%)	6 (28.6%)
**Staining intensity**						
Absent	3 (14.3%)	8 (38.1%)	7 (33.3%)	14 (66.7%)	1 (4.8%)	9 (42.9%)
Faint	2 (9.5%)	1 (4.8%)	5 (23.8%)	0 (0.0%)	7 (33.3%)	8 (38.1%)
Moderate	10 (47.6%)	4 (19.0%)	7 (33.3%)	3 (14.3%)	6 (28.6%)	2 (9.5%)
Strong	6 (28.6%)	8 (38.1%)	2 (9.5%)	4 (19.0%)	7 (33.3%)	2 (9.5%)
**Staining Score**						
0	3 (14.3%)	8 (38.1%)	7 (33.3%)	14 (66.7%)	1 (4.8%)	9 (42.9%)
1	0 (0.0%)	0 (0.0%)	3 (14.3%)	0 (0.0%)	0 (0.0%)	0 (0.0%)
2	1 (4.8%)	4 (19.0%)	3 (14.3%)	1 (4.8%)	2 (9.5%)	2 (9.5%)
3	1 (4.8%)	2 (9.5%)	3 (14.3%)	0 (0.0%)	0 (0.0%)	2 (9.5%)
4	4 (19.0%)	5 (23.8%)	3 (14.3%)	2 (9.5%)	3 (14.3%)	2 (9.5%)
5	2 (9.5%)	0 (0.0%)	1 (4.8%)	2 (9.5%)	2 (9.5%)	3 (14.3%)
6	4 (19.0%)	1 (4.8%)	0 (0.0%)	1 (4.8%)	6 (28.6%)	1 (4.8%)
7	6 (28.6%)	1 (4.8%)	1 (4.8%)	1 (4.8%)	7 (33.3%)	2 (9.5%)
